# Case of Somnambulism

**Published:** 1848-06

**Authors:** Nereus Mendenhall

**Affiliations:** Jamestown, Guilford county, N. C.


*Case of Somnambulism.* By Nereus Mendenhall, M. D., of Jamestown, Guilford county, N. C.

To the Editor of the Medical Examiner.

I have recently met with a case of somnambulism which has a bearing on the present doctrine of Phrenology.

The somnambulist is Miriam G., aged about 17 years. As usual in such cases, after retiring to bed, she will get up, dress herself, walk about the house, and converse freely. At the time alluded to, there was a light in the room. Her eyes were open, and their expression was intelligent. I asked her many questions in geography, which she answered promptly and correctly, though some of them were proposed in a manner designed to puzzle.

But the point to which attention is invited is this: A bible printed in small type was handed to her; she took it and *read*

*therein with facility, yet she failed to recognize the persons who were with her, though she knew some of them well.* She mistook comparative strangers for her own brothers; indeed in the many trials made with her in this respect, I am not aware that she was right in any instance, except one, when her attention was called to an article of dress, which of itself distinguished the person who wore it.

Now, Phrenology teaches that we have no knowledge of any operations of the mind, except those performed through the medium of the brain, and that of this, a particular part is employed exclusively, in each mental manifestation. Whatever may be the manifestation, it is invariably connected with, and made through one, and always the selfsame organ, as much so as that the bile is always secreted by the liver, the urine by the kidneys, &c. It also teaches that there is an organ of *form* which is defined to be "that mental power which takes cognizance of the shape or configuration of objects, and recollects them;" and we are told that one who has this organ large, notices and recollects the countenances of people, detects typographical errors, &c. Lyman Cobb is mentioned as a very accurate proof reader, since he had this organ large.

This, then, and no other, is the organ by which all *forms* are taken notice of—letters as well as persons. Thus speaks Phrenology.

Now, in the case under consideration, this organ (if such there be) was either awake or it was not awake. If *awake*, why did not the girl, when specially directed thereto, distinguish other persons from her own brothers? If the organ was not *awake*, how could she read fine print?

I propose these questions to be answered by those who may be able, having myself no interest to serve for or against Phrenology, except as it rests on the basis of truth or of error.

*Second month, 22d,* 1848.

